# Phylogeography of *Nasutitermes ephratae* (Termitidae: Nasutitermitinae) in neotropical region

**DOI:** 10.1038/s41598-022-15407-z

**Published:** 2022-07-08

**Authors:** Amanda de Faria Santos, Eliana Marques Cancello, Adriana Coletto Morales

**Affiliations:** 1 Instituto de Biociências, Letras e Ciências Exatas (IBILCE), Universidade Estadual Paulista (UNESP), São José do Rio Preto, SP Brazil; 2 Museu de Zoologia da Universidade de São Paulo (MZUSP), Universidade de São Paulo (USP), São Paulo, SP Brazil; 3 Faculdade de Ciências Agrárias e Veterinárias (FCAV), Universidade Estadual Paulista (UNESP), Jaboticabal, SP Brazil

**Keywords:** Genetics, Evolution, Evolutionary genetics, Population genetics

## Abstract

The neotropical region ranks third in the number of termites and includes five different families. Of these, Termitidae is the most diverse and includes the species *Nasutitermes ephratae*, which is widespread in the neotropics. To date, only one study has been published about phylogeography in neotropical termites (*N. corniger*). Here, we explored the population genetic patterns of *N. ephratae* and also evaluated the phylogeographical processes involved in the evolutionary history of the species. We used the mitochondrial genes 16S rRNA and COII as molecular markers: these were sequenced for 128 samples of *N. ephratae*. We estimated the genetic diversity and divergence time as well as the demography and genetic structure. We also performed an ancestral area reconstruction and a haplotype network. The results showed high genetic variability, recent demographic expansion, and strong genetic structure. A dispersal route for the species, that occurred in both directions between South and Central America, was inferred. The results emphasize a temporary separation between the South and Central America populations that affected the origin of the current Central America populations. These populations were formed from different phylogeographic histories.

## Introduction

The neotropics host a large diversity of species and habitats that arose from the complex geological history occurring along environmental and climatic changes. Evolutionary phenomena such as vicariance, dispersion, and extinction shaped the geographical distribution patterns of the species found in this region^1^.

The neotropical termite fauna ranks third in the number of species. Of the five neotropical termite families, Termitidae is the most diverse and includes the subfamily Nasutitermitinae with 171 species described in the neotropical region, including 67 in the genus *Nasutitermes*—corresponding to almost 40% of the number of species of the subfamily^2^.

Like the other Nasutitermitinae, the soldiers of this genus are characterized by a conic-shaped frontal projection with the opening of an exocrine gland located at the top. This gland produces substances used for defense against predators^3^.

**Figure 1:**
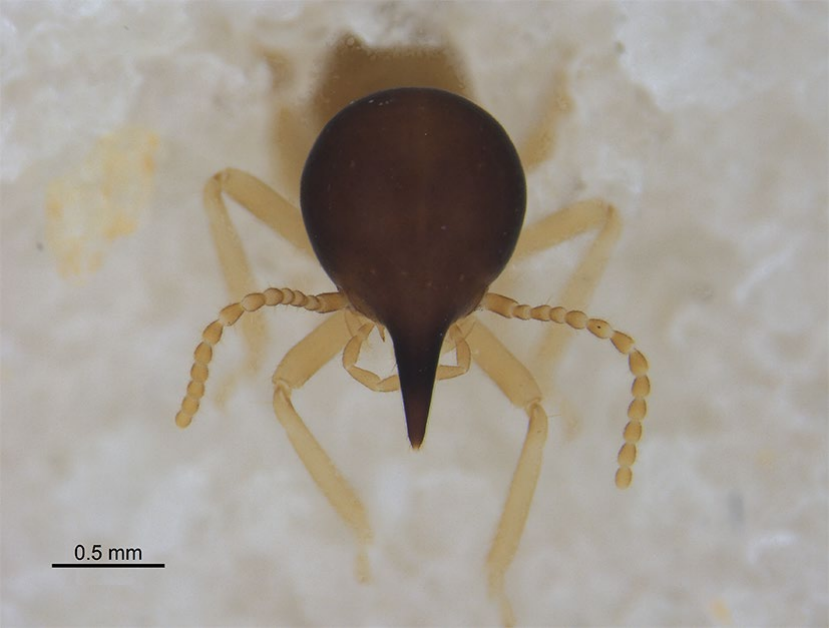
Soldier of *N. ephratae.*

*Nasutitermes ephratae* (Fig. 1) was described by Holmgren^4^ using alates and workers collected in Ephrata, Suriname. Banks^5^ described the soldiers using specimens of *N. creolina* collected in Panama. Later Snyder^6^ synonymized *N. creolina* with *N. ephratae*. The nests of this species are arboreal and show a light to dark brown coloration and a leathery surface as if they were enveloped. Internally, the nests are reinforced around the royal camara that harbors the queen and the king in the center of the nest (closer to the trunk or branch of the tree)^7^.

*Nasutitermes ephratae* is reconstructed close to *N. corniger* in various phylogenetic studies^8–11^. Both species are very common in the neotropics including most of Central and South America, e.g., Brazil, Bolivia, Colombia, Ecuador, French Guiana, Guyana, Peru, Suriname, and Venezuela^2,6,12–15^.

To date, only one phylogeographic study addressed neotropical termites, focusing on *N. corniger*^15^. The results showed high variability and strong genetic structure for the populations sampled. These were divided in haplogroups along its occurrence area associated with South American biomes. The authors also proposed a dispersal route for *N. corniger*, which would have left Central America towards South America, where the populations dispersed toward the eastern regions.

Phylogeographic and biogeographic studies help to explain how the species responded to geological and climatic changes over time. This fact highlights the importance of phylogeographic studies that present a variety of taxonomic groups, including insects, that have population and evolutionary dynamics different from other taxa^16^. Thus, the association of various phylogeographic data can help us to understand the evolutionary history of neotropical biota.

The main aim of this study was to explain the phylogeographical and historical processes that gave rise to population patterns of *N. ephratae* in the neotropics. For this purpose, we leveraged the mitochondrial molecular markers 16S rRNA and the subunit II of the code gene to cytochrome oxidase (COII). It is important to highlight that the mitochondrial DNA (mtDNA) have been extensively used for the investigation of phylogeographic issues as well as other evolutionary questions of a large variety of species, including termites^15,17–23^.

We performed demographic analyses, estimates of variability and genetic structure, analyses of divergence time, and ancestral area reconstruction. We also performed a haplotype network and proposed a dispersal route for the species.

## Methods

### Biogeographic units

The biogeographic units defined for the analyses are according to the neotropical regionalization in dominions proposed by Morrone^24^, as follows (considering the areas that were sampled): Antillean subregion (ANT) and Mesoamerican dominion (MES) in Central America; Pacific dominion (PAC) in Central and South America; Boreal Brazilian dominion (BOR), Chacoan dominion (CHA), Parana dominion (PAR), and south Brazilian dominion (SOU) in South America.

### Sampling

**Figure 2:**
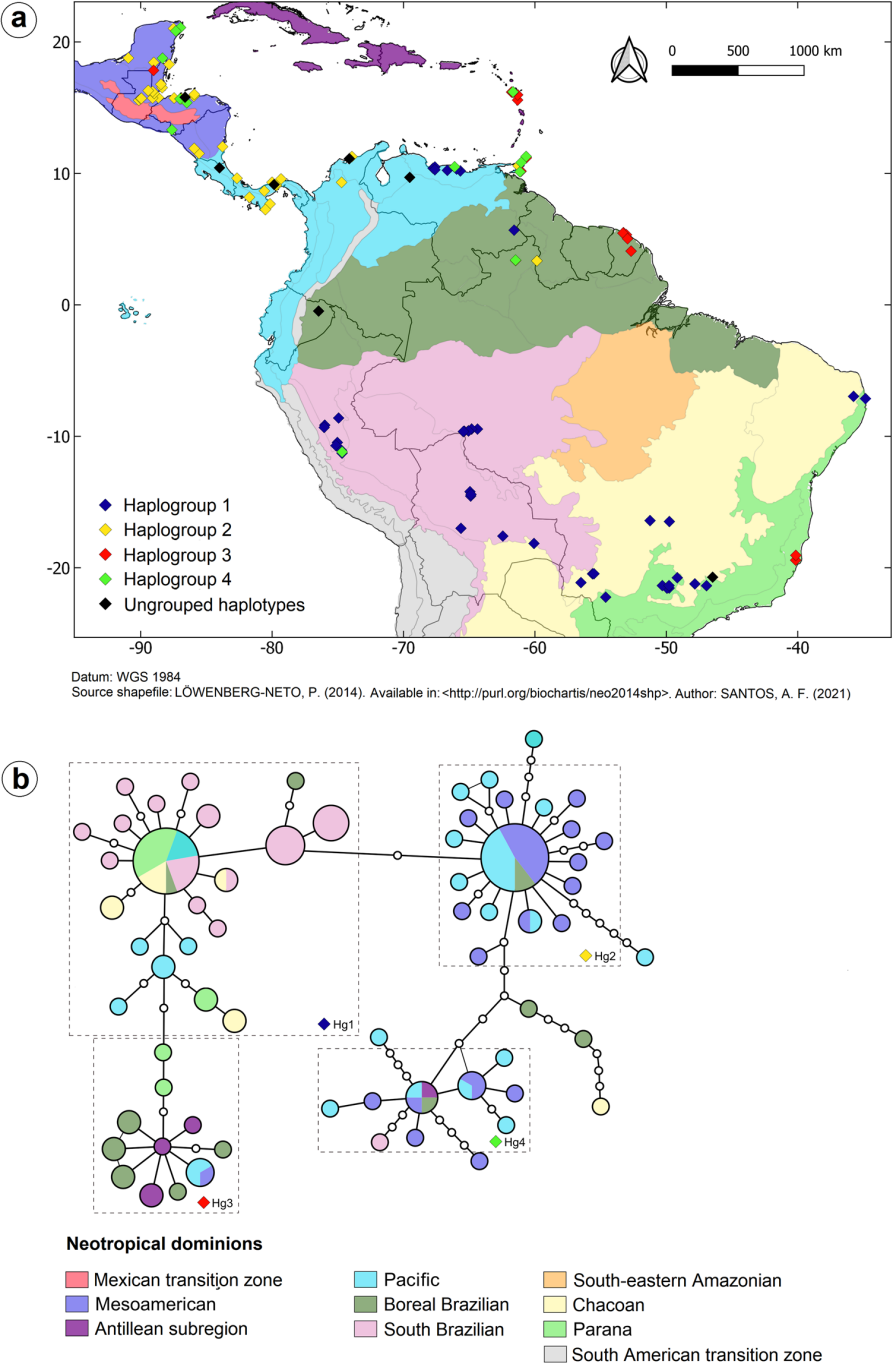
(**a**) Collection locations of the *N. ephratae* samples. The colors of the points are corresponding to the haplogroups; (**b**) Haplotype network generated using the concatenated sequences (16S + COII) of the *N. ephratae* samples. The white dots on the branches are indicating the mutational steps between the related haplotypes. The map (**a**) was generated by AFS using the software QGis v. 3.6.3 (http://qgis.org)^40^.

The samples of *N. ephratae* (Table 1; Fig. 2a) analyzed here have been obtained from the Isoptera collections of the *Museu de Zoologia da Universidade de São Paulo* (MZUSP) and the University of Florida (UF). The samples were collected in different campaigns and stored in 96% ethanol (MZUSP) and in 85% ethanol (UF) to better preservation of the DNA. Extracted DNA are stored in the Molecular Collection of the *Laboratório de Biologia Evolutiva* (LaBE) of the *Faculdade de Ciências Agrárias e Veterinárias*, UNESP (Jaboticabal, SP, Brazil). In addition to these samples, 12 nucleotide sequences of *N. ephratae* obtained in GenBank (public access) were included in the analyses, totaling 128 samples analyzed.

**Table 1:** Samples of *N. ephratae* analyzed with their respective collection location, geographical coordinates, sequencing of mtDNA regions and neotropical dominion.

Samples	Collection location, state (BR)/country	Geographical coordinates		Sequencing	Neotropical dominion
		Latitude	Longitude		
756	Bonito, MS, Brazil	− 21.1284	− 56.4957	16S/COII	CHA
757	Porto Velho, RO, Brazil	− 9.44788	− 64.811609	16S/COII	SOU
758	Porto Velho, RO, Brazil	− 9.44788	− 64.811609	16S/COII	SOU
759	Porto Velho, RO, Brazil	− 9.60887	− 65.376932	16S/COII	SOU
760	Porto Velho, RO, Brazil	− 9.44788	− 64.811609	16S	SOU
761	Porto Velho, RO, Brazil	− 9.44788	− 64.811609	16S/COII	SOU
762	Porto Velho, RO, Brazil	− 9.44788	− 64.811609	16S	SOU
763	Porto Velho, RO, Brazil	− 9.591526	− 65.05023	16S	SOU
765	Porto Velho, RO, Brazil	− 9.579086	− 65.05786	16S/COII	SOU
766	Porto Velho, RO, Brazil	− 9.591526	− 65.05023	16S/COII	SOU
767	Porto Velho, RO, Brazil	− 9.642215	− 65.446262	16S/COII	SOU
768	Porto Velho, RO, Brazil	− 9.591526	− 65.05023	16S/COII	SOU
770	Porto Velho, RO, Brazil	− 9.632081	− 65.438702	16S/COII	SOU
774	Porto Velho, RO, Brazil	− 9.45022	− 64.36745	16S/COII	SOU
775	Arceburgo, MG, Brazil	− 21.365503	− 46.94185	16S/COII	PAR
776	Promissão, SP, Brazil	− 21.545429	− 49.782324	16S/COII	PAR
777	Avanhandaga, SP, Brazil	− 21.554946	− 49.950317	16S/COII	PAR
778	Linhares, ES, Brazil	− 19.4225	− 40.1596	16S/COII	PAR
779	Coroados, SP, Brazil	− 21.356732	− 50.305391	16S/COII	PAR
780	Ipora, GO, Brazil	− 16.4124	− 51.2391	16S/COII	CHA
781	Aquidauana, MS, Brazil	− 20.4587	− 55.6164	16S/COII	CHA
782	Santa Bárbara, MG, Brazil	− 16.483	− 49.7686	16S/COII	CHA
783	Promissão, SP, Brazil	− 21.356201	− 49.79478	16S/COII	PAR
784	Guapiaçu, SP, Brazil	− 20.75824	− 49.165914	16S/COII	PAR
785	Ribeirão Preto, SP, Brazil	− 21.22689	− 47.826861	16S/COII	CHA
786	Palmeiras, MS, Brazil	− 20.4553	− 55.5053	16S/COII	CHA
787	Dourados, MS, Brazil	− 22.2373	− 54.6144	16S/COII	PAR
789	São João Batista, MG, Brazil	− 20.7176	− 46.4742	16S/COII	CHA
790	Sooretama, ES, Brazil	− 19.0554	− 40.1469	16S/COII	PAR
802	Rio Chico, Venezuela	10.32965	− 65.95991	16S/COII	PAC
807	Colon, Panama	9.12209	− 79.71566	16S	PAC
808	Chiquila, Mexico	21.02455	− 87.4977	16S/COII	MES
809	Las Quebradas, Honduras	15.38002	− 86.48891	16S/COII	MES
811	Alta Verapaz, Guatemala	15.56674	− 90.14269	16S/COII	MES
814	Minca, Colombia	11.1256	− 74.11972	16S/COII	PAC
820	Pipeline Road, Panama	9.12582	− 79.71581	16S/COII	PAC
821	Soberania Nat. Park, Panama	9.08148	− 79.66596	16S/COII	PAC
822	Ometepe, Nicaragua	11.51468	− 85.55514	16S/COII	MES
823	Izabal, Guatemala	15.75838	− 88.64599	16S/COII	MES
826	Francisco de Orellana, Ecuador	− 0.4708	− 76.45925	16S/COII	BOR
827	Minca, Colombia	11.11327	− 74.12861	16S/COII	PAC
830	Trinity Hills, Trinidad and Tobago	10.12008	− 61.13279	16S/COII	PAC
831	Englishman's Bay, Trinidad and Tobago	11.28833	− 60.66867	16S/COII	PAC
832	Aragua, Venezuela	10.49	− 67.61	16S	PAC
836	Coyolito, Honduras	13.31492	− 87.62271	16S/COII	MES
838	La Ceiba, Honduras	15.66692	− 87.00109	16S/COII	MES
842	Tayrona Nat. Park, Colombia	11.27731	− 73.92561	16S/COII	PAC
844	Maracay, Venezuela	10.27289	− 67.61113	16S/COII	PAC
847	Miranda, Venezuela	10.23373	− 66.66384	16S	PAC
849	Colon, Panama	9.57705	− 79.32218	16S/COII	PAC
850	Bluefields Naval Station, Nicaragua	12.03739	− 83.77062	16S/COII	PAC
853	Alta Verapaz, Guatemala	15.68823	− 89.98703	16S	MES
858	Henri Pittier Nat. Park, Venezuela	10.39418	− 67.75036	16S/COII	PAC
859	Grand Riviere, Trinidad and Tobago	10.83	− 61.044	16S/COII	PAC
861	Bolivar, Venezuela	5.683	− 61.583	16S	BOR
862	Satipo, Peru	− 11.28681	− 74.67691	16S/COII	SOU
864	Puerto Asese, Nicaragua	11.90023	− 85.92898	16S/COII	MES
866	Lancetilla Botanical, Honduras	15.73359	− 87.45594	16S/COII	MES
867	Alta Verapaz, Guatemala	15.71261	− 89.94968	COII	MES
868	Ansela Frais, Guadalupe	15.97567	− 61.31552	16S/COII	ANT
871	Past Mojoriver, Belize	16.09314	− 88.9702	16S/COII	MES
872	Cochabamba, Bolivia	− 16.99937	− 65.62736	16S/COII	SOU
873	Yacaumbu Nat. Park, Venezuela	9.69985	− 69.52694	16S/COII	PAC
883	Mahaut, Guadalupe	16.18723	− 61.7735	16S/COII	ANT
886	Rio Blanco Nat. Park, Belize	16.22892	− 89.09382	16S/COII	MES
887	Sierra de Cochis, Bolivia	− 18.14974	− 60.06951	16S/COII	SOU
890	Aripo Savannah, Trinidad and Tobago	10.59667	− 61.2075	16S	PAC
892	Rio Negro, Peru	− 11.18987	− 74.66985	16S/COII	SOU
893	Colon, Panama	9.32286	− 80.00095	16S/COII	PAC
894	Los Santos, Panama	7.25147	− 80.50834	16S/COII	PAC
895	Quintana Roo, Mexico	21.09713	− 86.96915	16S	MES
896	Pico Bonito Lodgetrail, Honduras	15.68348	− 86.90016	16S/COII	MES
900	Heredia, Costa Rica	10.4254	− 84.0022	16S/COII	PAC
901	Maya Point, Belize	16.52775	− 88.36321	16S/COII	MES
902	Tauri Mennonite site, Bolivia	− 17.58995	− 62.44228	16S/COII	SOU
907	Bajo Pichanaqui, Peru	− 11.06414	− 74.71955	16S/COII	SOU
910	Quintana Roo, Mexico	20.83018	− 87.32672	16S/COII	MES
915	Heredia, Costa Rica	10.4254	− 84.0022	16S/COII	PAC
918	San Javier, Bolivia	− 14.54909	− 64.88964	16S/COII	SOU
922	Alto Cacazu old forest, Peru	− 10.70755	− 75.14109	16S/COII	SOU
924	Los Santos, Panama	7.67865	− 80.15967	16S/COII	PAC
925	Laguna Bacalar, Mexico	18.76662	− 88.33867	16S	MES
926	Capiro Nat. Park, Honduras	15.88046	− 85.94997	16S/COII	MES
927	Peten, Guatemala	16.30402	− 89.42172	16S/COII	MES
930	Limon, Costa Rica	9.63252	− 82.67172	16S/COII	PAC
933	San Pedro, Bolivia	− 14.2126	− 64.94026	16S/COII	SOU
937	Pte. Bermudez, Peru	− 10.46894	− 75.03005	16S/COII	SOU
940	San Jose, Mexico	18.4409	− 89.00258	16S/COII	MES
949	El Coco, Venezuela	10.18912	− 65.6721	16S	PAC
950	Arena Forest, Trinidad and Tobago	10.57657	− 61.27255	COII	PAC
952	Campoverde, Peru	− 8.60854	− 74.93628	16S/COII	SOU
953	Lajas de Tole, Panama	8.1874	− 81.72511	16S/COII	PAC
955	San Jose, Mexico	18.296	− 87.83277	16S/COII	MES
956	Laguna Guaimoreto, Honduras	16.01322	− 85.91839	16S/COII	MES
957	Izabal, Guatemala	15.73636	− 89.091	16S	MES
961	Cockscomb Nat. Park, Belize	16.78049	− 88.45901	16S/COII	MES
964	Higuerote Beach, Venezuela	10.50282	− 66.11221	16S	PAC
967	Tingo Maria Cacao, Peru	− 9.32776	− 76.03557	16S/COII	SOU
969	Cocle, Panama	8.66907	− 80.59178	16S/COII	PAC
977	San Pedro, Bolivia	− 14.4239	− 64.86053	16S/COII	SOU
981	Rushville, Trinidad and Tobago	10.16633	− 61.05433	16S/COII	PAC
982	Tingo Maria, Peru	− 9.14974	− 75.99233	16S/COII	SOU
983	Barro Colorado Is., Panama	9.1521	− 79.8464	16S/COII	PAC
985	Chicbul, Mexico	18.78033	− 90.93848	16S/COII	MES
986	Sambo Creek, Honduras	15.79585	− 86.62127	16S/COII	MES
988	Inra, Guadalupe	16.20458	− 61.6666	16S	ANT
990	Francisco de Orellana, Ecuador	− 0.4708	− 76.45925	16S	BOR
991	Cienega La Batea, Colombia	9.32242	− 74.70874	16S/COII	PAC
1078	João Pessoa, PB, Brazil	− 7.13445	− 34.84602	16S/COII	PAR
1079	João Pessoa, PB, Brazil	− 7.13445	− 34.84602	16S/COII	PAR
1080	Areia, PB, Brazil	− 6.962804	− 35.754688	16S/COII	CHA
1081	Areia, PB, Brazil	− 6.962804	− 35.754688	16S/COII	CHA
1084	Amajari, RR, Brazil	3.3778	− 61.46444	COII	BOR
1085	Amajari, RR, Brazil	3.405	− 61.47333	16S/COII	BOR
1086	Bonfim, RR, Brazil	3.35111	− 59.846944	COII	BOR
1087	Bonfim, RR, Brazil	3.35111	− 59.846944	COII	BOR
BZ15^a^	Rio Bravo conservation area, Belize	17.836799	− 89.019253	16S (AY623088)	MES
DM59^a^	St. Andrew, Dominica	15.58	− 61.320032	16S (AY623086)	ANT
GU113^a^	Basse− Terre, Guadalupe	16.166813	− 61.664298	16S (AY623089)	ANT
NOU1^a^	Nouragues, French Guiana	4.087108	− 52.680544	16S (KF724740)/COII (KC630996)	BOR
NOU2^a^	Nouragues, French Guiana	4.087108	− 52.680544	16S (KF724739)/COII (KC630996)	BOR
PAT2^a^	Patagai, French Guiana	5.48	− 53.26	16S (KF724741)/COII (KC630997)	BOR
PAT3^a^	Patagai, French Guiana	5.48	− 53.26	16S (KF724739)/COII (KC630998)	BOR
PAT4^a^	Patagai, French Guiana	5.48	− 53.26	16S (KF724738)/COII (KC630999)	BOR
ROC1^a^	Rocoucoua, French Guiana	5.455818	− 53.304559	16S (KF724738)/COII (KC630995)	BOR
RSE1^a^	RouteSaint-Élie, French Guiana	5.335233	− 53.035583	16S (KF724739)/COII (KC631000)	BOR
ST18^a^	PetitSaut, French Guiana	5.03333	− 52.95	16S (KX816700)/COII (KX816672)	BOR
TT644^a^	PinfoldBay, Tobago	11.188005	− 60.657997	16S (AY623087)	PAC
Samples					128
16S sequences					123
COII sequences					108
Concatenated sequences					103

ANT: Antillean subregion; BOR: Boreal Brazilian dominion; CHA: Chacoan dominion; MES: Mesoamerican dominion; PAC: Pacific dominion; PAR: Parana dominion; SOU: South Brazilian dominion.

^a^Nucleotide sequences obtained in GenBank.

### Laboratory procedures

The total DNA was extracted from the head of an individual per colony following Liu and Beckenbach^25^ protocol, which includes phenol–chloroform for the extraction and 100% and 70% ethanol for the DNA washing. The amplification of the 16S rRNA and COII (gene regions of mtDNA) was performed by PCR following the conditions described in Supplementary Table S1. The PCR reaction was composed by 0.6 pmol/μL of each primer (forward and reverse)^26–28^, PCR Master Mix (Promega) 1x, nuclease free water, and 2.4 ng/μL of target DNA. The PCR product was purified using the Wizard^®^ SV Gel and PCR Clean-Up System (Promega) according to the manufacturer’s instructions, followed by Sanger sequencing in ABI 3730 XL DNA Analyzer (Applied Biosystems) automatic sequencer.

### Data analysis

#### Genetic diversity and neutrality tests

The nucleotide sequences were read in Chromas Lite v. 2.6.5 (Technelysium Ltd., 2005). The sequences of 16S were aligned using Mafft v. 7^29,30^ and the sequences of COII were aligned using Geneious v. 7.1.9 (https://www.geneious.com) by ClustalW, both followed by inspection by eye. Geneious also was used to concatenate the 16S and COII sequences of the samples in which both regions could by sequenced.

To quantify the genetic diversity, the following parameters were estimated using DnaSP v. 6^31^: number of polymorphic sites (S), nucleotide diversity (π), average number of nucleotide differences (k), and haplotype diversity (Hd). In addition to these parameters, the value of θ-W per sequence was estimated using Arlequin v. 3.5.1.2^32^. This software also was used to perform the Fu’s *Fs*^33^ and Tajima’s *D*^34^ neutrality tests—these tests were performed for the entire sample set and, separately, for each neotropical dominion sampled. The other neutrality tests Fu and Li’s F* and D* and Achaz Y*^35^ were performed using DnaSP v. 6^31^.

The nucleotide composition of the sequences and the pairwise genetic distances (among individuals, among dominions and within dominions) were estimated using MEGA-X^36^ (the model Kimura 2-parameter^37^ was used to estimate the genetic distances). The genetic distances and the neutrality tests were estimated for both the concatenated sequences (16S + COII) and the single genes (16S or COII). Only the single genes were considered to estimate the genetic diversity indexes.

### Haplotype network

The haplotype network was generated with the concatenated sequences (16S + COII) using TCS v. 1.21^38^, that uses parsimony method to establish the relationship among the haplotypes, with 95% of connection limit. The haplogroups were defined based on the shape of the network, considering the distance among the haplotypes and among the tips and central haplotypes of the group. The network was colored using tcsBU^39^ considering the frequency of the haplotype in each neotropical dominion sampled.

The map showing the distribution of the samples, colored according to the observed haplogroups, was performed using QGIS v. 3.6.3^40^ based on the datum WGS 1984 and on the shapefile of the neotropical region developed by Löwenberg-Neto^41^, considering the regionalization proposed by Morrone^24^.

### Genetic structure and Mantel test

The Analysis of Molecular Variance (AMOVA) was performed using Arlequin v. 3.5.1.2^32^ to assess the possibility of genetic structure among the sampled populations. Using the concatenated sequences, tree constructions were defined for this analysis: (i) an AMOVA was performed considering the haplogroups of the haplotype network; (ii) the second AMOVA was performed separating the samples in seven groups according to their neotropical dominion (ANT, BOR, CHA, MES, PAC, PAC, and SOU); (iii) the third AMOVA was also performed considering the dominions as groups, but regrouping them into two major groups: Central America (ANT, MES, and northern PAC) and South America (BOR, CHA, southern PAC, PAR, and SOU). The F indexes, calculated by AMOVA, range from 0 to 1 and indicate high differentiation above 0.25 and moderately high differentiation between 0.15 and 0.25^42^.

Even to evaluate the inference of genetic structure, we performed an analysis of DNA clustering using the package rhierBAPS^43,44^ implemented in R v. 4.0.1^45^. The results were obtained for both the concatenated sequences and the single genes. The graphs showing the geographical distribution of each genetic cluster (considering the concatenated sequences results) were obtained using Microsoft Excel (2008).

The Mantel test was performed using the package vegan^46^, implemented in R v. 4.0.1, considering the Pearson’s correlation as method with a number of permutation equal to 10,000. We inputted the concatenated sequences and the geographical coordinates of each sample to perform this analysis.

### Analysis of divergence time and ancestral area reconstruction

We estimated the divergence time among the samples of *N. ephratae* relying on Bayesian inference. The tree was generated in BEAST v. 2.6.3^47^ using strict clock and chain length equal to 90 million. Two partitions were included in the analysis, the first containing the 16S sequences and the second containing the COII sequences. The tree models (Fossilized Birth Death Model)^48^ and the clock models for both partitions were linked, and the substitution model TrN + I + G were applied for 16S and COII^49^. The selection of the best-fit model of nucleotide substitution was done based on the results of jModelTest^50^ considering the lower values of BIC.

The Bayesian inference was calibrated with the ages of four fossil records, following Heath, Huelsenbeck, and Stadler instructions^48^: *Valditermes brenanae*^51^, 136.4 to 130 million of years ago (My); *Nanotermes isaace*^52^, 56 to 47.8 My; *Nasutitermes electrinus*^53^, 23.03 to 15.97 My; and *Atlantitermes caribea*^54^, 20.44 to 13.82 My. Besides the fossil records, the origin dates estimated by Bourguignon^55^ for Termitidae (54 My), Nasutitermitinae (26.2–19.4 My), and *Nasutitermes* (22.6–16.4 My) were used as an additional calibration to improve de analysis estimation (Supplementary Figure S2; Supplementary Table S2). For the MRCA (most recent common ancestor) priors we used exponential distribution. As outgroups, we included the species *Mastotermes darwiniensis* (Mastotermitidae), *Amitermes dentatus* (Termitidae: Termitinae), *Atlantitermes snyderi* (Termitidae: Nasutitermitinae), and *Nasutitermes longinasus* (Termitidae: Nasutitermitinae).

The MCMC trace files generated by Bayesian inference were viewed and analyzed using Tracer v. 1.7.1^56^ to check the values of Effective Sample Size (ESS > 200). The trees were resampled using the BEAUti v. 2.6.3 application “Full To Extant Tree Combiner”^47^ to remove the fossil taxa of the topology, keeping only the extant species. The best tree was annotated by TreeAnnotator v. 2.6.3^57^ with 10% burn-in, and it was viewed and draw using FigTree v. 1.4.4^58^.

The tree topology, as well as the divergence times estimated by this analysis, were used for the ancestral area reconstruction analysis, which was performed using the package BioGeoBEARS^59^ in R v. 4.0.1^45^. We defined seven occurrence areas for this analysis, according to the neotropical dominions which were sampled (ANT, BOR, CHA, MES, PAC, PAR and SOU). We tested the models DEC, DIVALIKE and BAYAREALIKE with and without *j* (i.e., six models tested). The best-fit model was selected based on the lower values and higher weights of AIC and AICc.

## Results

### Genetic diversity and demographic inferences

We obtained 123 nucleotide sequences for 16S [396 base pairs (bp)] and 108 for COII (742 bp) within the samples, totaling 103 specimens with both mtDNA genes sequenced. The nucleotide composition of the concatenated sequences corresponds to 40.27% of adenine (A), 25.35% of thymine (T), 21.20% of cytosine (C), and 13.19% of guanine (G). The mean genetic distance among the samples was 0.011 for the concatenated sequences, 0.007 for the 16S sequences, and 0.015 for COII sequences (the pairwise genetic distances are presented in Supplementary Tables S3, S4, and S5). After estimating the genetic distances among and within the dominions, higher values were obtained for the BOR-MES and BOR-PAC pairwise comparisons (Table 2). The mean genetic distance within dominions was 0.008.

**Table 2:** Genetic distance values estimated (a) among the dominions and (b) within dominions.

(a) Genetic distance among the neotropical dominions sampled						
	ANT	BOR	CHA	MES	PAC	PAR
BOR	0.011					
CHA	0.010	0.011				
MES	0.013	0.016	0.013			
PAC	0.013	0.016	0.013	0.013		
PAR	0.010	0.010	0.003	0.012	0.012	
SOU	0.010	0.011	0.004	0.012	0.012	0.003

(b) Genetic distance within the Neotropical dominions sampled						
Dominion	Genetic distance					
ANT	0.008					
BOR	0.012					
CHA	0.005					
MES	0.012					
PAC	0.014					
PAR	0.001					
SOU	0.003					

ANT: Antillean subregion; BOR: Boreal Brazilian dominion; CHA: Chacoan dominion; MES: Mesoamerican dominion; PAC: Pacific dominion; PAR: Parana dominion; SOU: South Brazilian dominion.

We found 66 haplotypes considering the concatenated sequences: 41 haplotypes (Hd = 0.890) for 16S and 53 haplotypes (Hd = 0.932) for COII (Table 3a), indicating high genetic variability for the populations sampled. High values also were found for the number of polymorphic sites (S) of both mtDNA genes (24 for 16S and 87 for COII). The COII synonym sites showed the higher value of nucleotide diversity (π), which was 200 times higher than the value observed for the non-synonym sites (Table 3a). The 16S π value was lower than the COII π value, which tends to present more polymorphism.

**Table 3:** (a) Genetic diversity indices and (b and c) neutrality tests performed for the *N. ephratae* populations.

(a) Genetic diversity							
Gene	Num. of sequences	Num. of sites	h (Hd)^a^	S^a^	k^a^	π^a^	θ-W/seq
16S	123	396	41 (0.890)	24	3.851	0.00972	3.793 ± 1.216
COII	108	742	53 (0.932)	87	11.560	0.01551	16.354 ± 4.312
		syn = 154.8 n.syn = 529.2 n.cod = 58				syn = 0.069 n.syn = 3.5e^−4^	

(b) Neutrality tests for the whole sample set (16S + COII)^b^							
Fu/Li D*	Fu/Li F*	Achaz Y*	Fu’s *Fs*	Tajima’s *D*			
− 2.190 (n/s)	− 2.089 (n/s)	0.02162	**− 26.072** (p = 0.00)	− 0.884 (n/s)			
**− 5.833** (p < 0.02)	**− 5.333** (p < 0.02)	− 1.65154	**− 24.454** (p = 0.00)	− 1.225 (n/s)			
**− 4.962** (p < 0.02)	**− 4.584** (p < 0.02)	− 1.31284	**− 26.431** (p = 0.00)	− 0.915 (n/s)			

(c) Neutrality tests for dominions (16S + COII)							
Test	Neotropical dominion						
	ANT	BOR	CHA	MES	PAC	PAR	SOU
Fu’s *Fs*	**− 2.370** (p = 0.02)	**− 10.558** (p = 0.000)	**− 12.470** (p = 0.000)	**− 26.545** (p = 0.000)	**− 25.996** (p = 0.000)	**− 34.028** (p = 0.000)	**− 28.194** (p = 0.000)
Tajima’s *D*	− 1.145 (n/s)	− 0.349 (n/s)	− 1.513 (n/s)	− 0.479 (n/s)	− 0.006 (n/s)	− 1.128 (n/s)	− 1.201 (n/s)
Fu’s *Fs*	2.996 (n/s)	**− 7.832** (p = 0.002)	**− 4.656** (p = 0.004)	**− 12.963** (p = 0.000)	**− 13.563** (p = 0.001)	**− 14.439** (p = 0.000)	**− 26.495** (p = 0.000)
Tajima’s *D*	0.000 (n/s)	0.062 (n/s)	− 1.527 (n/s)	1.247 (n/s)	− 0.428 (n/s)	0.336 (n/s)	**− 2.267** (p = 0.002)
Fu’s *Fs*	**− 2.370** (p = 0.02)	**− 10.558** (p = 0.000)	**− 4.063** (p = 0.01)	**− 26.629** (p = 0.000)	**− 26.022** (p = 0.000)	**− 13.607** (p = 0.000)	**− 28.194** (p = 0.000)
Tajima’s *D*	− 1.145 (n/s)	− 0.349 (n/s)	**− 1.591** (p = 0.004)	− 0.647 (n/s)	− 0.176 (n/s)	− 0.077 (n/s)	− 1.200 (n/s)

Syn: synonym; n.syn: non-synonym; n.cod.: no coding site; n/s: not significant (p > 0.05). ANT: Antillean subregion; BOR: Boreal Brazilian dominion; CHA: Chacoan dominion; MES: Mesoamerican dominion; PAC: Pacific dominion; PAR: Parana dominion; SOU: South Brazilian dominion.

Significant values are highlighted in bold.

^a^h: number of haplotypes; Hd: haplotype diversity; S: number of polymorphic sites (including aligned gaps for 16S); k: average number of nucleotide difference; π: nucleotide diversity.

^b^For Fu and Li’s D* and F* and for Achaz Y*, we considered only the samples with both mitochondrial genes sequenced.

Regarding to the neutrality tests performed with the whole sample set (Table 3b), the Tajima’s *D* value was not significant. This means that there is not enough evidence to discriminate between demographic expansion or population bottlenecks from these indices. The negative and significant value of Fu’s *Fs,* i.e., the most sensitive among the neutrality tests performed^60^, suggest demographic expansion for the populations, which is also suggested from the negative value of Achaz Y*. Regarding the neutrality tests performed per dominion (Table 3c), the negative and significant values of Fu’s *Fs* also indicate demographic expansion for all the dominions Only the value of Tajima’s *D* for CHA was significantly negative. The results of neutrality tests performed for each gene are presented in Supplementary Table S6.

### Haplotype network

We generated a haplotype network to reconstruct the relations among the 66 haplotypes found for *N. ephratae* (Fig. 2b). We observed four haplogroups (Hg) composed, in general, by haplotypes close each other and originated from a more frequent central haplotype. Haplotypes that were very distant from the central haplotype of the haplogroup (i.e., that present many mutational steps) were not grouped. The star shape of the haplogroups suggests recent demographic expansion events for the populations analyzed.

The network also showed a clear geographic differentiation among South American populations composed of haplogroup 1 and Central American populations composed mostly of haplogroups 2 and 4 (Fig. 2b; Supplementary Table S7). The haplogroup 3 is formed mainly by populations located in northern South America (BOR) and in the Antillean islands. Haplogroup 3 also includes haplotypes from eastern Brazil (PAR – Espírito Santo, Brazil), which are intermediaries between this haplogroup and the haplogroup 1. The geographical distribution of the haplogroups is also showed in Fig. 2a.

### Population structure and isolation by distance

**Figure 3:**
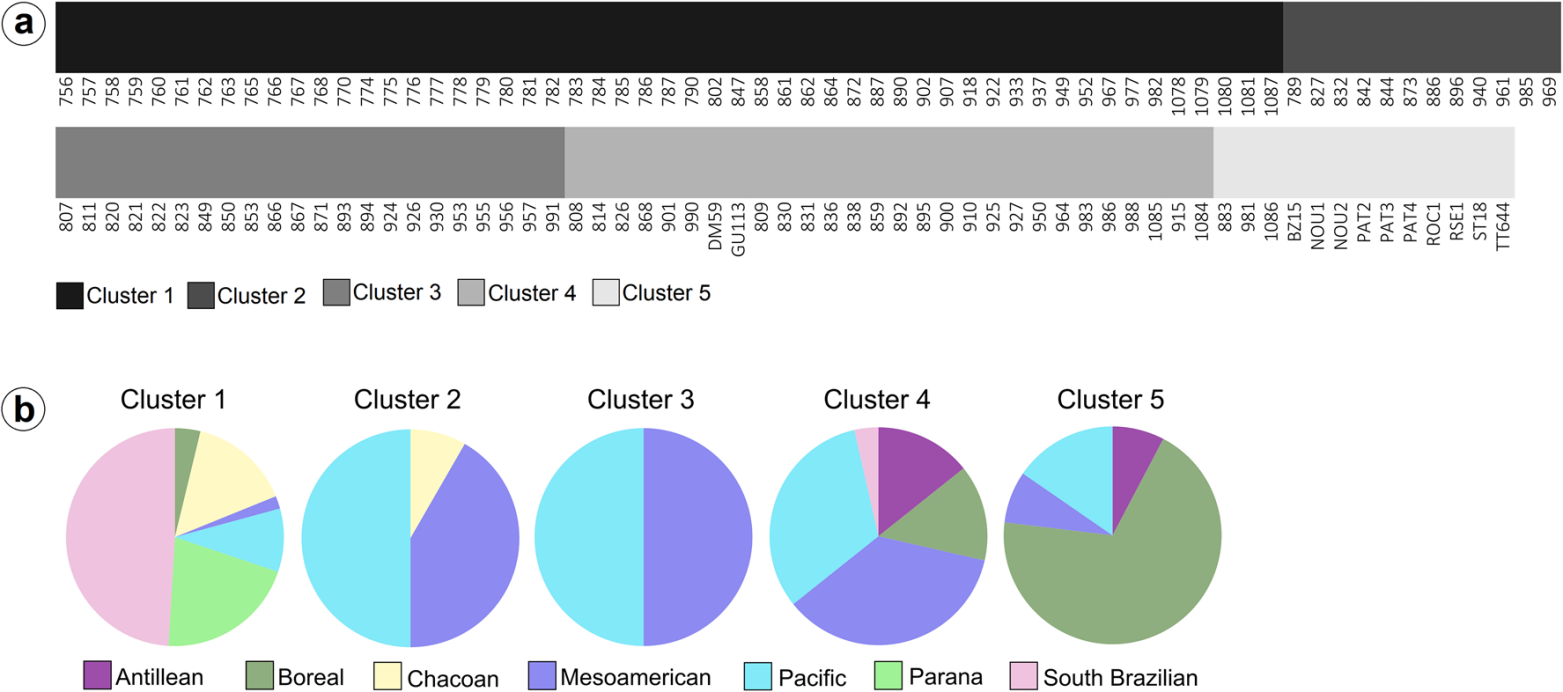
(**a**) Results of the analysis of clustering (rhierBAPS) performed with the concatenated sequences; (**b**) Pie charts generated from the frequency of the dominions in each cluster recovered by rhierBAPS;

The inference of population structure in *N. ephratae* was verified from the results of the AMOVA (Table 4) and rhierBAPS (Fig. 3). Regarding the AMOVA among haplogroups (Table 4a), the F_ST_ value was significantly very high indicating strong differentiation among the haplogroups established in the network (Fig. 2b). A high F_ST_ was also observed in the AMOVA among dominions (Table 4b), showing strong genetic differentiation among the dominions sampled. In the AMOVA among continents (Table 4c), the high F_CT_ also indicates strong differentiation among the populations located in different continents while a moderate differentiation can be observed among the dominions within continents, as showed by F_SC_.

**Table 4:** Results obtained for the Analysis of Molecular Variance (AMOVA).

Source of variation	Percentage of variation (%)	Fixation indices
**(a) Among haplogroups**		
Among haplogroups	54.69	F_ST_ = **0.547** (p = 0.000)
Within haplogroups	45.31	–
**(b) Among dominions**		
Among dominions	31.07	F_ST_ = **0.310** (p = 0.000)
Within dominions	68.93	–
**(c) Among continents**		
Among continents	25.91	F_CT_ = **0.259** (p = 0.02)
Among dominions within continents	16.82	F_SC_ = **0.227** (p = 0.000)
Within dominions	57.27	F_ST_ = **0.427** (p = 0.021)

Significant values are in [bold].

The rhierBAPS results (Fig. 3) showed five genetic clusters (represented by different shades of gray in Fig. 3a) for the *N. ephratae* samples. The samples of the cluster 1 are distributed in South America; the samples of cluster 2 are distributed in northern South America (Venezuela and Colombia in southern portion of PAC) and Central America (MES dominion). Cluster 3 includes only samples from Central America (MES and northern portion of PAC), and cluster 4 includes samples from BOR, ANT, and mainly from PAC (northern and southern portions) and MES. Cluster 5 includes samples from BOR and southern portion of PAC (Fig. 3b). These results also suggest strong genetic differentiation among the populations located in South and Central America.

Mantel’s test was performed to verify the existence of isolation by distance and resulted in a significantly positive *r* value (0.1197; p = 0.0005), thus indicating that there is a positive correlation between the genetic distance and the geographic distance, i.e., the genetic distances can increase as geographic distance between populations increase.

### Divergence time and ancestral area reconstruction

**Figure 4:**
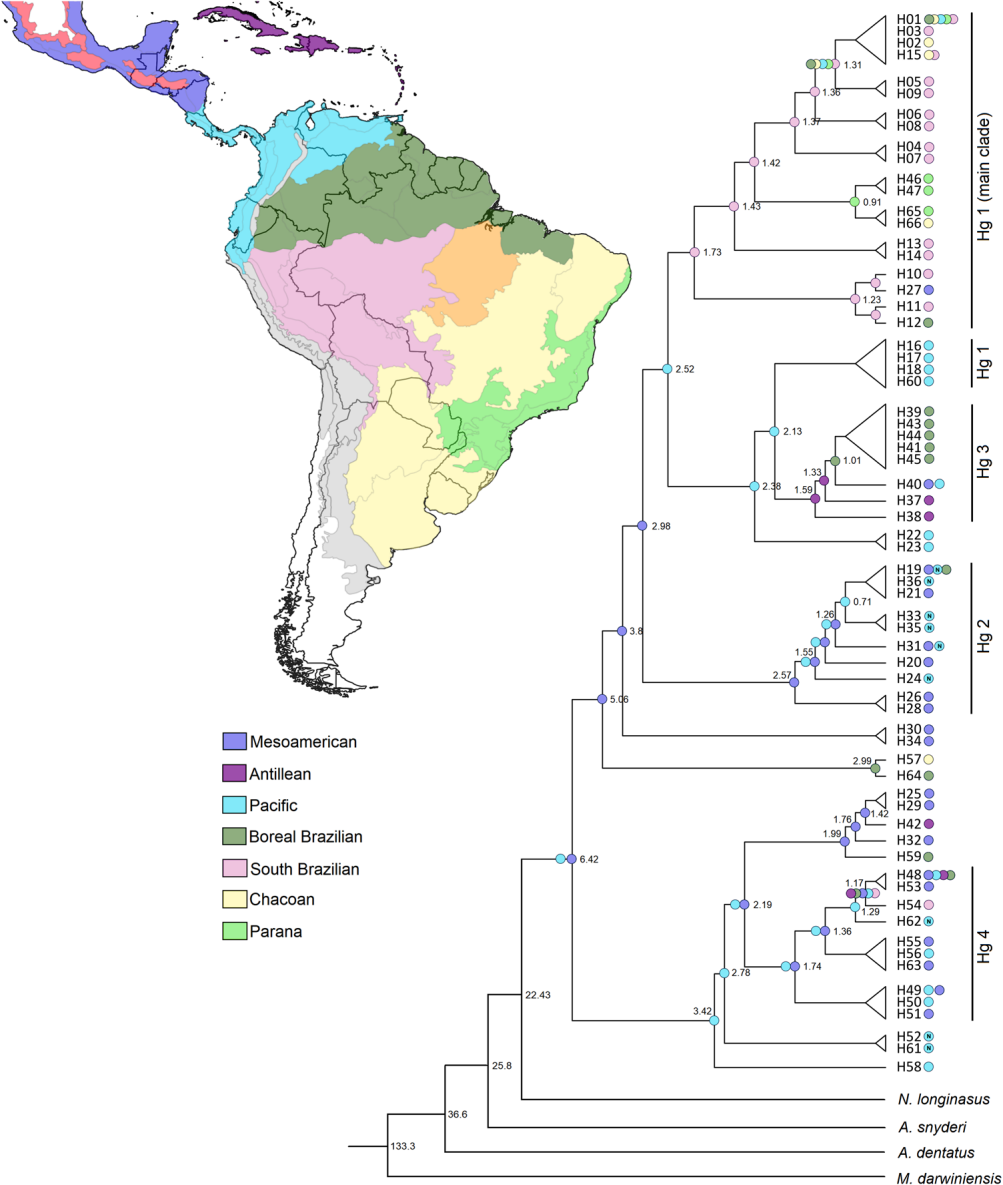
Bayesian inference tree generated using the haplotypes of *N. ephratae*, associated to the results of the ancestral area reconstruction. The numbers near to the nodes correspond to the estimated divergence times in million of years (My). The “N” inside the PAC circles (close to the taxa names) are indicating that the respective haplotype was observed in the northern portion of PAC dominion, located in Central America; PAC circles without the “N” are indicating the haplotypes from southern portion of PAC dominion, located in South America. The posterior probabilities and the 95% HPD intervals for this BI are showed in Supplementary Fig. S2. The map was generated by AFS using the software QGis v. 3.6.3 (http://qgis.org)^40^.

The divergence times of the *N. ephratae* populations were estimated from a Bayesian inference (BI) analysis performed using the haplotypes of the 16S and COII sequences (Fig. 4; Supplementary Fig. S2). According to the tree obtained, three important cladogenetic events seem to have given rise to the *N. ephratae* populations analyzed. The first event (6.42 My) separated the Hg 4 + H25-H59 clade (that includes samples from Central America and northern South America) from the other clades – the ancestral node of this clade was dated to 2.19 My by the analysis and the Hg 4 clade was dated to 1.74 Ma. The second event (2.98 My) separated the Hg 1 clades + Hg 3 clade from the Hg 2 clade, which ancestral node was dated to 2.57 My. The last one includes only specimens distributed in Central America (except for one sample of H19 found in BOR). The third event (2.52 My) caused the divergence among the Hg 1 main clade (formed by South American populations) from the Hg 1 + Hg 3 clade (formed by samples from northern South America and Antillean islands). The ancestral nodes of the Hg 3 and the Hg 1 main clade were dated to 1.59 My and 1.73 My, respectively.

For the ancestral area reconstruction, the best-fit model selected, DEC + *j*, presented AIC and AICc equal to 211.7 and 212.4 with weights equal to 0.85 and 0.84, respectively. The results showed that the common ancestor of all the clades was distributed in MES and PAC dominions and were dispersed posteriorly to the other areas (Fig. 4; Supplementary Fig. S3).

## Discussion

Data on the genetic diversity obtained for the *N. ephratae* populations showed high genetic variability for the species, as evidenced by the large number of haplotypes in relation to the total number of samples as well as by the high haplotype diversity. As expected, the nucleotide diversity observed for the COII sequences was greater than the nucleotide diversity found for the 16S. Furthermore, the π value obtained for the COII synonyms sites was higher than the π value for the non-synonym sites (Table 3a).

The high genetic variability observed for *N. ephratae* seems not to be widely shared among the populations, as highlighted by the AMOVA results (Table 4), which showed strong genetic structure for the species. That is, there are a lot of genetic variants that are exclusive of one or few regions suggesting low or moderated gene flow among distant populations. This limitation in gene flow may have occurred due to isolation by distance as confirmed by Mantel’s test. The clustering analysis results also corroborated the population structure inference because five clusters with a distribution limited to adjacent dominions were recovered. Cluster 1 shows a larger geographic distribution and occurs in all South American dominions. Great genetic variability with haplotypes little shared among distant geographic regions was also observed very similar to *N. corniger*^15^.

Regarding to the demographic history of the *N. ephratae* populations, the neutrality tests (Table 3b,c), specifically Fu’s *Fs*, indicated demographic expansion for the species (considering the whole sample set) and particularly for each neotropical dominion sampled. This also can be inferred from the star shape of the haplogroups in the haplotype network, thus suggesting that a lot of descendent haplotypes had recently risen from the ancestor haplotypes located in the center of the haplogroups.

The haplotype network (Fig. 2b) also showed four haplogroups presenting clear differences about the geographical distribution of the haplotypes. Haplogroup 1 is entirely composed of haplotypes from the South America dominions while haplogroups 2 and 4 are mostly formed by Central American haplotypes. Some haplotypes included in haplogroup 4 can also be found in northern South America (Venezuela and Trinidad and Tobago) but were not often observed at latitudes below this. Haplogroup 3 is composed of haplotypes from northern South America (Trinidad and Tobago and French Guiana) and from the Antillean islands (Dominica and Guadeloupe).

These groups were also recovered by the clustering analysis (Fig. 3a) except for a few differences among them. In general, the results of the analyses are linked as following: haplogroup 1 corresponds to cluster 1; haplogroup 2 corresponds to cluster 3; haplogroup 3 corresponds to cluster 5; and haplogroup 4 corresponds to cluster 4.

**Figure 5:**
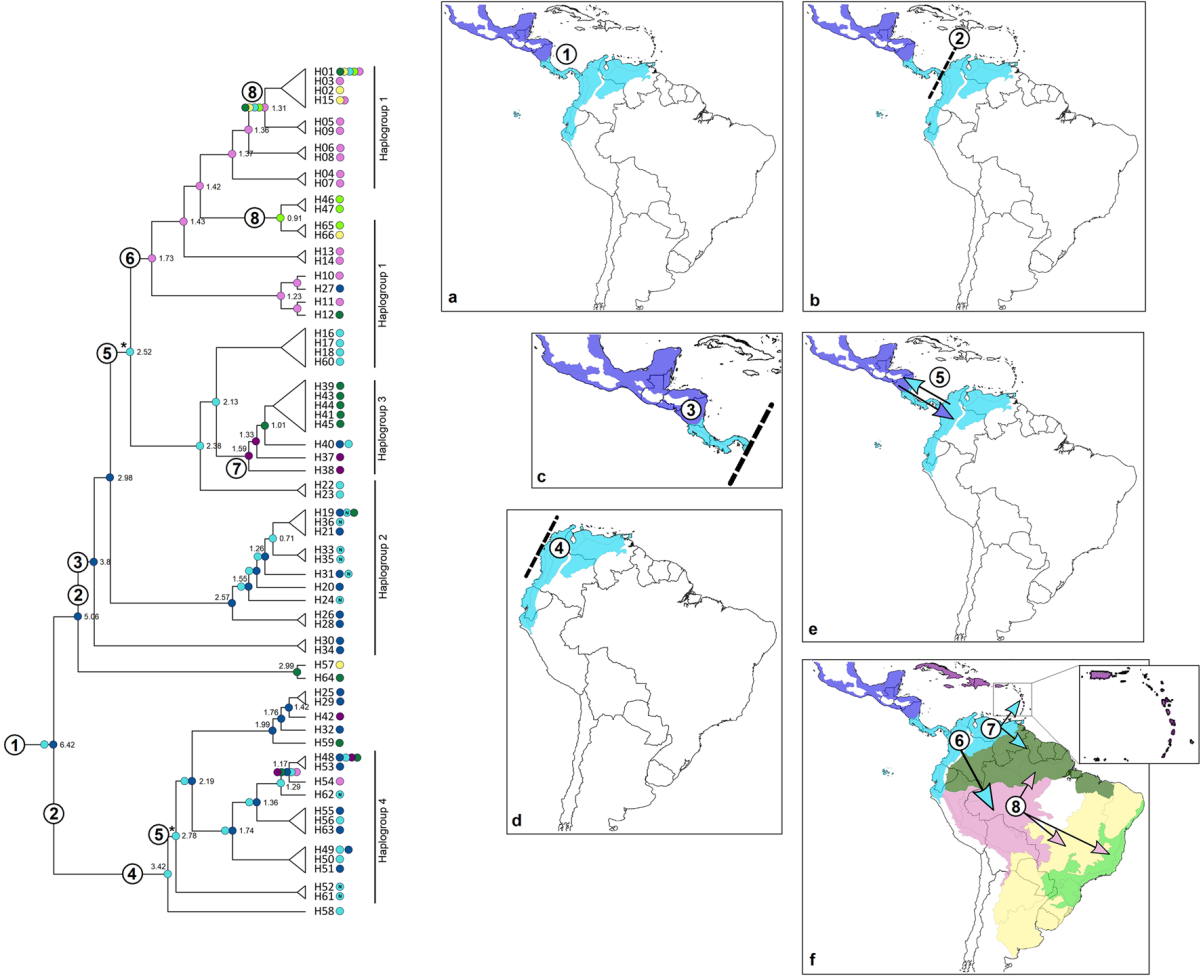
Dispersal route inferred for *N. ephratae* based on the results obtained in the ancestral area reconstruction. The arrows are indicating the direction of the dispersion. *Reconnection between the *N. ephratae* populations of South and Central America. The map was generated by AFS using the software QGis v. 3.6.3 (http://qgis.org)^40^.

The BI analysis (Fig. 4; Supplementary Fig. S2) shows that some nodes of the tree presented posterior probabilities below 0.50 possibly due to the difficulties of the algorithm, implemented in BEAST, in solving datasets containing very similar sequences, which occurs in many intraspecific analyses^61^. Despite this, large clades of this tree include haplotypes from the same haplogroup (Fig. 4), helping to support the phylogenetic inferences raised by BI. Observing the topology of the tree, the haplotype network, and the ancestral ranges reconstructed, we inferred dispersal events and then proposed a dispersal route for the *N. ephratae* populations (Fig. 5).

The ancestral populations distributed in MES and PAC (“1”; Fig. 5a) suffered a temporary separation that split the populations of South America (southern PAC) from the Central American (northern PAC + MES) populations (“2”; Fig. 5b). The ancestor of the haplogroups 1, 2, and 3 occurred in MES during this separation (“3”; Fig. 5c). This leads to the origin of haplogroup 2 that was restricted to Central America (northern PAC + MES). After the reconnection of the *N. ephratae* populations (indicated with an asterisk in Fig. 5), the Mesoamerican ancestor dispersed to the South American portion of PAC (“5”; Fig. 5e) from where there was a new dispersion to SOU (“6”, Fig. 5f) and to ANT-BOR (“7”, Fig. 5f). This last dispersion gave rise to haplogroup 3 composed mainly of French Guiana and Antillean populations. The ancestral populations of the haplogroup 1 that had arisen in SOU were widely dispersed to CHA and PAR (Atlantic Forest lato sensu) reaching to BOR and southern PAC (“8”, Fig. 5f), but remaining limited to South America.

Still during the temporary separation between the South and Central America *N. ephratae* populations, the South American ancestor of the haplogroup 4 arose in southern PAC (“4”; Fig. 5d). After the populations’ reconnection, there was a dispersal from southern PAC to northern PAC and to MES originating as the late ancestor of haplogroup 4 and clade H25-A59, which were restricted to these dominions (“5”; Fig. 5e).

Based on the dispersal route proposed, we inferred that the *N. ephratae* populations currently distributed in Central America arose from distinct dispersal events. This becomes clearer when we also observe the geographic distribution of haplogroups 2 and 4 (Fig. 2) whose occurrence areas overlap, but who have different genetic groups. That is, even though these haplogroups are distributed in the same area, they are results of different evolutionary events. The ancestors of these haplogroups possibly diverged during a temporary split among the ancestral *N. ephratae* populations from South and Central American between the late Pliocene and early Pleistocene (around 5.06 and 2.78 My; Figs. 4 and 5). Haplogroup 2 originated from populations that were restricted to Central America during this split while the haplogroup 4 originated after the reconnection perhaps arising from populations that were dispersed from the northern South America (southern PAC) to Central America (northern PAC + MES). The same direction of dispersion (South America to Central America) was also identified for the ant species *Neoponera villosa*^16^ dated from 0.46 to 0.28 My; earlier dispersions have been observed for *N. ephratae*.

Haplogroup 1 is exclusively South American and arose from a Mesoamerican ancestor that dispersed to the northern South America and then to the SOU dominion. Therefore, the dispersal events of the *N. ephratae* populations among Central and South America occurred in both senses, thus shaping the genetic and phylogeographic patterns observed here.

Although the causes responsible for the temporary split between the South and Central American *N. ephratae* population are not clear, some hypotheses can be proposed. A population isolation caused by the geographic distance between the populations may have led to this split: The occurrence of isolation by distance was suggested by the Mantel’s test. The loss of distribution area could also have caused this effect in the populations. In this way, the reconnection of the populations could have occurred due to the demographic expansion that was detected by the neutrality tests and can be suggested from the star shape of the haplogroups in the haplotype network.

Geological and/or geographic factors could also help to explain this split. Following this approach, it is possible that the separation is related to the tertiary's tectonic and paleographical reorganization movements (in the late Pliocene), which led to the emergence of barriers and changes in dispersal routes in South America^62^. Specifically, this split between the *N. ephratae* populations may have been caused by the momentaneous effects of the elevation of the Panama isthmus. These effects would have lasted between 4.6 and 2.6 My^62,63^—a date close to the one estimated for the separations detected (5.06 to 2.78 My). Moreover, the early Quaternary climate changes (early Pleistocene) was characterized by temperature and dryness oscillations in the continents and was also impacted the adaptability and the migration of species and populations^62^.

Although dispersion by water (inside flotsam carried by ocean or river currents or ferried by vessels) helps to explain dispersion patterns for termite species^55,64,65^, it is more likely that the paths taken by the populations of *N. ephratae* were overland, which makes the PAC dominion an obligatory passage for the dispersal of populations between South and Central America. This dominion harbor peculiarly haplotypes from all haplogroups and present a higher value of intra-dominion genetic distance (Table 2b). These features help to infer the PAC intermediary position for the *N. ephratae* dispersions.

The dispersal route traced for the populations analyzed here are very similar to the dispersal route observed for *N. corniger* species^15^ including the dispersion from Central to South America, the eastward dispersion on South America lands, and the late occupation of Atlantic Forest. Nevertheless, the dispersal from South to Central America and the temporary split among the population have not yet been detected for *N. corniger* unlike *N. ephratae*. However, it is important to note that only the 16S mitochondrial marker was used for the *N. corniger* analyses^15^. The addition of other markers can lead to a more robust comparison among the population patterns of the two species.

Crews and Esposito^66^ also studied dispersal routes and identified that South America is most probably the origin of most of the Caribbean arthropod fauna including *N. ephratae*, *N. corniger*, and other species of the genus *Nasutitermes*. These data contradict the inferences raised by Santos et al.^15^ although there are some important methodological differences among the studies. Crews and Esposito^66^ analyzed 18 species of *Nasutitermes* using the same mtDNA genes used here as molecular markers (16S + COII). The dispersal route proposed herein is according to the inferences made by Crews and Esposito^66^ because the *N. ephratae* Antillean populations arose from a South America ancestor according to the ancestral area reconstruction. Specifically, for these island populations, it is possible that the dispersion from northern South America to the Antilles occurred via flotsam or floating wood carried to the islands by oceanic currents. This kind of dispersion has already been detected for Caribbean termite species^65^. Most of the islands’ termite species analyzed here are compose of haplogroup 3 together with samples from French Guiana (BOR dominion) and Trinidad and Tobago (located in the transition between BOR and PAC). This data suggests high genetic similarity among populations of these areas—although a larger sampling can better clarify the relations among the two dominions.

Regarding the BOR dominion, we found that the samples from this area have haplotypes typically found in other areas, which stayed distant from each other in the haplotype network (except for the haplotypes from French Guiana). This distance was also observed in BI. Some authors argue that the Amazonian biota (composed of parts of BOR and SOU dominions and the southeastern Amazon dominion—the latter was not sampled herein) have unnatural biogeographic origins^67–71^. As a speculation, this hypothesis could help clarify the existence of haplotypes that are genetically distant from each other in Amazonian localities that are geographically close to each other. However, we point that it is necessary to include a greater sampling of this region to clearly detect the genetic population patterns and the evolutionary events involving *N. ephratae* in this dominion.

In general, there are many questions to be explored about the phylogeographic processes of the South American and neotropical species especially for termites whose studies are still new. Better sampling of *N. ephratae* and/or including new molecular markers in future studies, as well as addressing phylogeographic issues of other species, can help solidify the inferences made in this work and can expand the understanding of the evolutionary history of the group and of the neotropics.

## Conclusions

This study considered most area where *N. ephratae* occurs in the neotropics. It was possible to make important inferences about the general panorama of the evolutionary history of the species in this region although a broader sampling, especially from central-eastern South America, could better clarify some phylogeographic patterns. Our data also showed similarities on the population and dispersal patterns among *N. ephratae* and *N. corniger*. Here, it is possible to speculate that both species responded similarly to the biogeographic processes that have occurred in the neotropics although new comparative studies may better answer this question. In summary, this study offers important contributions to the understanding of biogeographic and phylogeographic issues in the neotropics especially evolutionary studies of termites and other insects.

## Supplementary Information

Supplementary Figure S1.

Supplementary Figure S2.

Supplementary Figure S3.

Supplementary Tables.

## Data Availability

DNA sequences: Genbank accessions numbers OL830473–OL830583 (16S) and OL830584–OL830683 (COII).
