# Supplementary figures and images for: Phylogeography of Nasutitermes ephratae (Termitidae: Nasutitermitinae) in neotropical region
Source: Sci Rep. 2022 Jul 8;12:11656. doi: 10.1038/s41598-022-15407-z (PMC9270401; doi:10.1038/s41598-022-15407-z)

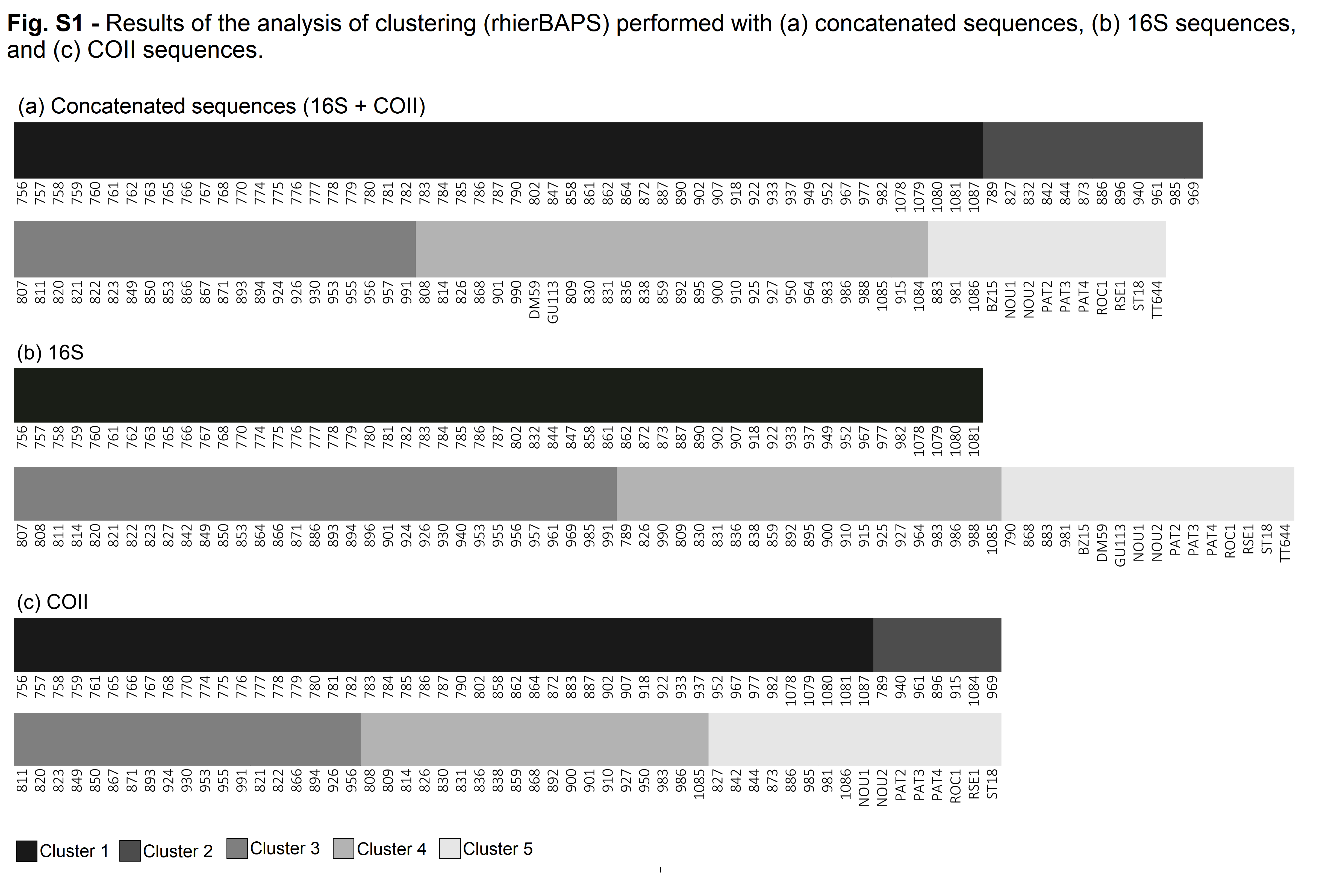

Supplement: Supplementary file 1 — Supplementary Figure S1. [file 41598_2022_15407_MOESM1_ESM.png]

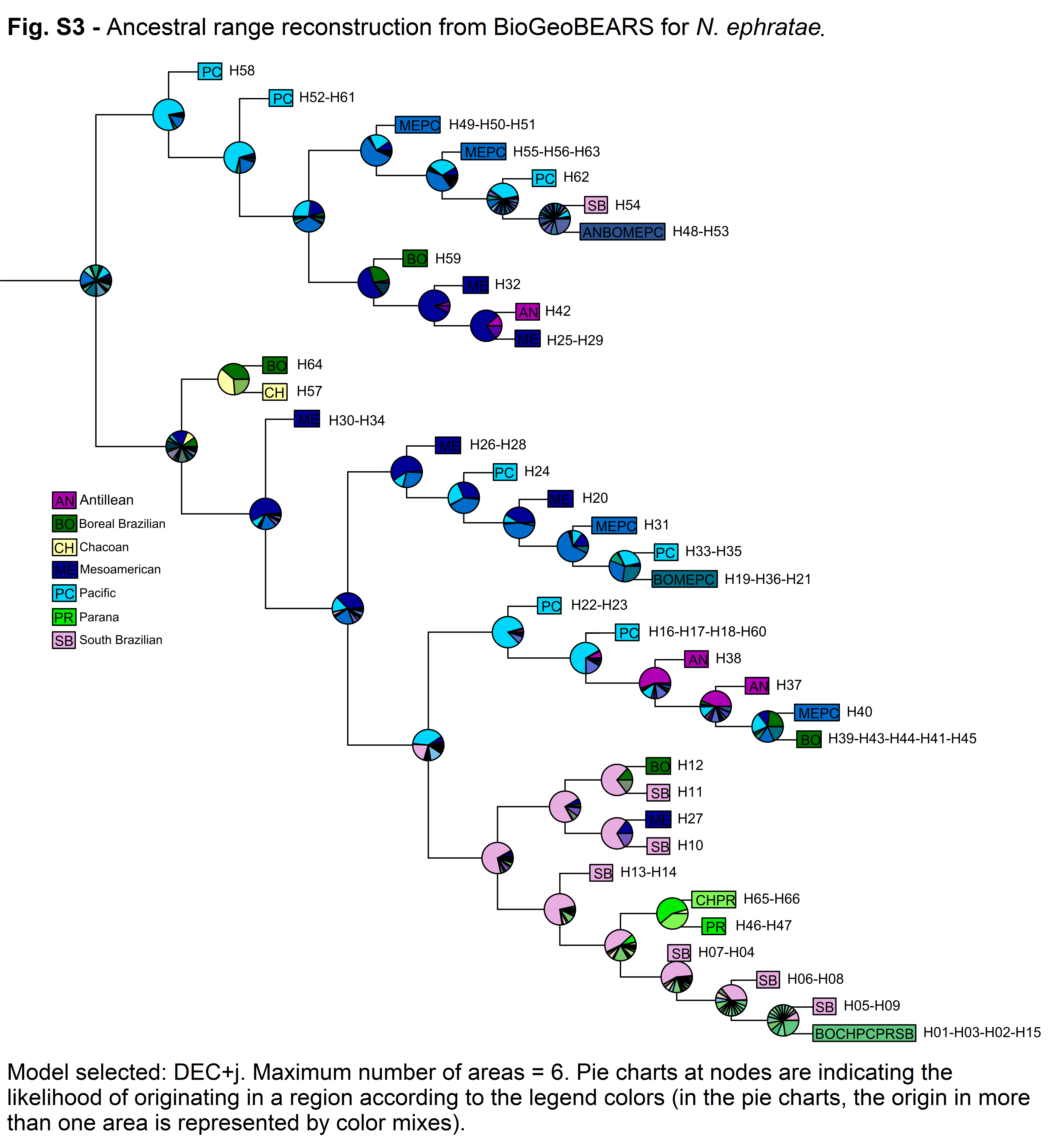

Supplement: Supplementary file 3 — Supplementary Figure S3. [file 41598_2022_15407_MOESM3_ESM.png]
